# Outbreak of hepatitis A virus infection in Taiwan, June 2015 to September 2017

**DOI:** 10.2807/1560-7917.ES.2019.24.14.1800133

**Published:** 2019-04-04

**Authors:** Wan-Chin Chen, Po-Hsun Chiang, Yu-Hsin Liao, Lin-Ching Huang, Ying-Jung Hsieh, Chu-Ming Chiu, Yi-Chun Lo, Chin-Hui Yang, Jyh-Yuan Yang

**Affiliations:** 1 Office of Preventive Medicine, Centers for Disease Control, Ministry of Health and Welfare, Taipei City, Taiwan; 2 PoloWang Bio. Inc, New Taipei City, Taiwan; 3 Center for Diagnostics and Vaccine Development, Centers for Disease Control, Ministry of Health and Welfare, Taipei City, Taiwan; 4 Division of Acute Infectious Diseases, Centers for Disease Control, Ministry of Health and Welfare, Taipei City, Taiwan; 5 Office of Deputy Director-General, Centers for Disease Control, Ministry of Health and Welfare, Taipei City, Taiwan

**Keywords:** hepatitis a, outbreaks, human immunodeficiency virus, HIV, sexually transmitted infections

## Abstract

The Taiwan Centers for Disease Control (CDC) were notified of increasing acute hepatitis A (AHA) in June 2015. Serum and/or stool from AHA patients and sewage samples were tested for hepatitis A virus (HAV). We defined outbreak cases as AHA patients with illness onset after June 2015 and with an HAV sequence less than 0.5% different from that of the TA-15 outbreak strain. We analysed characteristics and food exposures between outbreak and non-outbreak cases between January 2014 (start of enhanced surveillance) and February 2016. From June 2015 to September 2017, there were 1,563 AHA patients with a median age of 31 years (interquartile range (IQR): 26–38); the male-to-female ratio was 8.8 and 585 (37%) had human immunodeficiency virus (HIV) infection. TA-15 was detected in 82% (852/1,033) of AHA patients, and 14% (74/540) of sewage samples tested since July 2015. Infection with the TA-15 strain was associated with having HIV, sexually transmitted infections (STI), recent oral-anal sex and men who have sex with men (MSM). The Taiwan CDC implemented an HAV vaccine campaign starting from October 2016 where 62% (15,487/24,879) of people at risk were vaccinated against HAV. We recommend HAV vaccination for at-risk populations and continuous surveillance to monitor control measures.

## Introduction

Acute hepatitis A (AHA) is a disease caused by hepatitis A virus (HAV) that spreads through person-to-person contact or through contaminated food or water. In high-income countries where the prevalence of anti-HAV antibodies is low, infection usually occurs in susceptible adults, and transmission among travellers to endemic regions, men who have sex with men (MSM) and people who inject drugs (PWID) has been reported [1]. Because of genetic variability, comparison of HAV nucleotide sequences can identify genetic relatedness between different strains and provide useful information in outbreak investigations [2].

In the 1970s, the prevalence of HAV infection was high in Taiwan and most people had been exposed to or infected with HAV in childhood [3]. As highest prevalence of HAV was observed in indigenous townships because of inadequate water, sanitation and hygiene infrastructure in 1990s, Taiwan launched targeted HAV vaccination among children living in indigenous townships and surrounding areas starting in June 1995, covering 2% of the overall population [4]. According to the nationwide surveillance, HAV infection decreased significantly, from 663 AHA patients (annual incidence 2.96/100,000) in 1995 to 117 (annual incidence 0.5/100,000) in 2014. The highest incidence shifted from children and adolescents younger than 20 years to young adults and travellers to endemic countries; between 2010 and 2014, 96 to 139 AHA cases were reported annually, and the average male-to-female ratio was 1.3 [4,5]. The re-emergence of HAV among non-immunised populations is raising concerns as outbreaks of AHA among MSM have recently been reported in Europe and America [6,7].

## Outbreak detection

In June 2015, a considerable increase in reports of AHA infection was noted in Taiwan’s National Notifiable Diseases Surveillance System (NNDSS). Of the 133 AHA patients reported from June to December 2015, 47 (35%) were co-infected with human immunodeficiency virus (HIV); of these HIV patients, 46 were infected with identical HAV strain. Before 2015, only two AHA patients with HIV co-infection had been reported, in 2007 and 2011 [8]. The Taiwan Centers for Disease Control (CDC) coordinated an intra-agencies team to determine the outbreak scale, identify the at-risk population and propose control measures. 

Here we describe Taiwan’s epidemiological and virological investigations and the control measures that were implemented during the outbreak.

## Methods

### Surveillance of acute hepatitis A in Taiwan

AHA has been listed in the Communicable Diseases Control Act as a nationally notifiable disease in Taiwan since 1999. Physicians are expected to notify health authorities of AHA patients within 24 h of diagnosis, based on clinical assessment or positive HAV serology. In the NNDSS, a probable AHA patient is defined as a patient with a positive anti-HAV IgM result, and a confirmed AHA patient is defined as a symptomatic patient with a positive anti-HAV IgM result in addition to jaundice or alanine aminotransferase (ALT) ≥ 100 U/L. All reported AHA patients are investigated by health authorities using a semi-structural questionnaire to collect information on demographics, food or water exposures, travel history, oral-anal sex and injection drug use during the 60 days before illness onset. Confirmed AHA patients would be classified as locally acquired or imported infections based on the travel and exposure history obtained.

In March 2013, a slight increase in AHA reports of locally acquired and imported infections was observed. Beginning in 2014, the Taiwan CDC implemented enhanced surveillance by testing of human and environmental samples and through hypothesis-generating questionnaire interviews to identify the HAV outbreak [5]. Serum or stool samples of AHA patients were routinely sent to the national laboratory for virus detection and sequencing (Supplement); since March 2016, one in 10 specimens from AHA patients with HIV coinfection has been tested and sequenced. Patients with locally acquired AHA were re-interviewed by trained interviewers from the Taiwan CDC, using a hypothesis-generating questionnaire adapted from the Oregon Questionnaire [9].

### In-depth investigation, January 2014 to February 2016

We defined outbreak-associated cases as probable or confirmed AHA patients with illness onset after June 2015 and with an HAV sequence that differed by less than 0.5% from that of the outbreak strain. History of sexually transmitted infections (STI, including syphilis, gonorrhoea and shigellosis), HIV infection and mode of HIV transmission were ascertained by linking to the NNDSS and national HIV surveillance systems [10,11].

Using the hypothesis-generating questionnaire, we interviewed AHA patients reported before March 2016 and collected additional information on social venues and an open question regarding sexual behaviours within the 2 months before illness onset.

For each AHA patient, we defined syphilis, gonorrhoea or shigellosis as a recent infection if these STI were diagnosed between 12 months before and 10 days after AHA notification. We compared demographics, outcomes, presence of or recent STI, risk behaviours and food exposures between outbreak and non-outbreak (patients with HAV sequence ≥ 0.5% different from that of the outbreak strain) AHA cases diagnosed during the period from January 2014 to February 2016, using univariate analysis and Epi Info 7.1.5.2 software.

### Environmental surveillance

Environmental surveillance of sewage was set up in compliance with the World Health Organization (WHO) strategy plan of the Global Polio Eradication Initiative in Taiwan in 2012 [12]. We collected sewage specimens twice a month from the inlet collector canals of 10 wastewater treatment plants (WTP) across Taiwan. We started testing for HAV in sewage samples in July 2015 following an increase in AHA reports (Supplement).

### Phylogenetic analysis

CLUSTAL W was used to align the HAV VP1-2A genome sequences [13]. The evolutionary history was inferred using the general time reversible model, the best-fit substitution model of the maximum likelihood method. The phylogenetic tree was drawn to scale, with branch lengths reflecting the number of substitutions per site. We analysed fragments of 460 nt of the HAV VP1-2A genome sequences. Evolutionary analyses were conducted using MEGA6.

Bootstrap values were derived from 1,000 replicates [14]. All reference sequences used in this study were obtained from GenBank (accession numbers: AB618531, EF207320, AF357222, AF485328, HQ822086, AB623053, AB020565, AB020567, AB020568, EU131373, M14707, AF268396, AF314208, M20273, AY644676, AY644670, AB258387, JQ655151, AJ299464, DQ991029, EU011791, FJ360735 and DQ991030). The simian hepatitis A virus sequence with accession number SHVAGM27 was used as an outgroup.

### Ethical statement

This investigation was conducted in response to a public health emergency and was exempt from institutional review board approval.

## Results

### Hepatitis A outbreak in Taiwan

**Figure 1:**
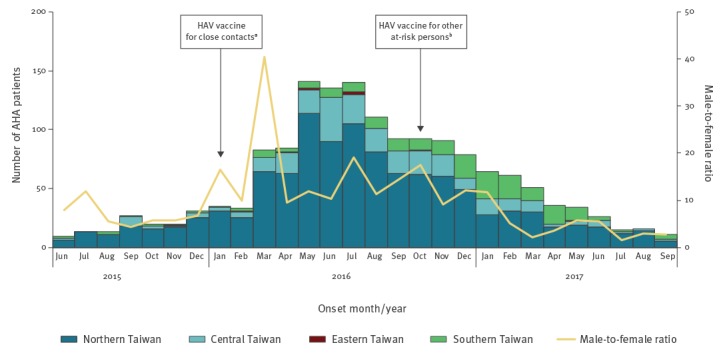
*. Number of confirmed acute hepatitis A patients, by month of onset and region of residence, Taiwan, June 2015–September 2017 (n = 1,563)

AHA: acute hepatitis A; HAV: hepatitis A virus.

From June 2015 to September 2017, the NNDSS received 1,563 reports of confirmed AHA patients (Figure 1). The median age was 31 years (range: 1–92 years; interquartile range (IQR): 26–38), the male-to-female ratio was 8.8, 1,424 cases (91%) involved locally acquired infection and 585 cases (37%) had reported HIV infection. The number of confirmed AHA patients peaked during the period from May to July 2016. Most patients (n = 1,345, 86%) resided in six metropolitan cities, and the residential region gradually shifted from northern Taiwan to southern Taiwan from late 2016 (Figure 1). Compared with the 170 AHA reported during the same period between 2012 and 2014, this outbreak represented an almost nine-fold increase.

Northern Taiwan: Yilan County, Keelung City, Taipei City, New Taipei City, Taoyuan City, Hsinchu City, Hsinchu County, Miaoli County, Kinmen County and Lienchiang County. Central Taiwan: Taichung City, Changhua County, Nantou County, Yunlin County, Chiayi City and Chiayi County. Southern Taiwan: Tainan City, Kaohsiung City, Pingtung County and Penghu County. Eastern Taiwan: Hualien County and Taitung County.

^a^ Close contacts included the household members and sexual partners of acute hepatitis A patients.

^b^ At-risk persons included individuals born after 1977, who were living with HIV or newly diagnosed with syphilis or gonorrhoea.

**Figure 2:**
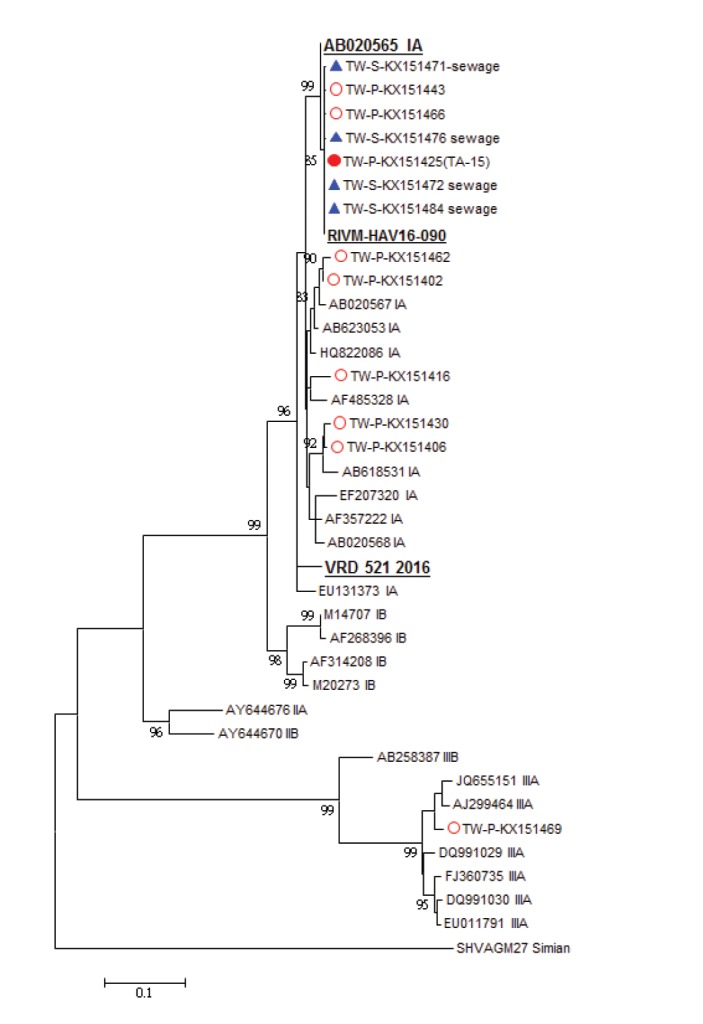
Phylogenetic tree of the hepatitis A virus VP1-2A region (460 nt), maximum likelihood method, Taiwan, June 2015–September 2017 (n = 69)

**Figure 3:**
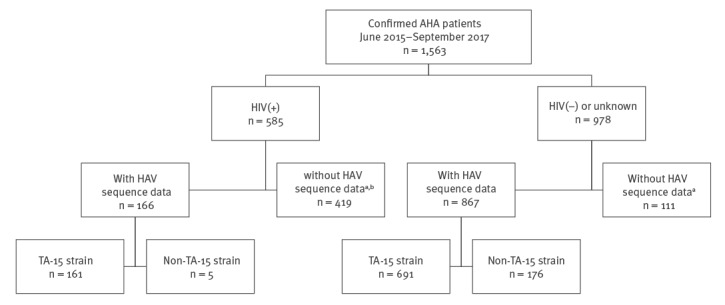
Acute hepatitis A patients with and without HIV co-infection and hepatitis A virus sequence results, Taiwan, June 2015–September 2017 (n = 1,563)

AHA: acute hepatitis A; HIV: human immunodeficiency virus; HAV: hepatitis A virus.

^a^ ‘Without HAV sequence data’ included insufficient specimens submitted for HAV sequencing (n = 32), negative HAV PCR results (n = 89), poor HAV sequence quality (n = 4) and AHA patients with HIV co-infection reported after March 2016 (n = 405).

^b^ One of every 10 specimens from AHA patients with HIV co-infection has been tested and sequenced since March 2016.

We identified a new circulating HAV strain named TA-15 (GenBank accession number: KX151425, subgenotype IA) in June 2015 (Figure 2). Of the 1,033 confirmed patients with available VP1-2A genome sequences, 852 (82%) were infected with HAV strain TA-15 (Figure 3). Among the 852 outbreak-associated cases, the median age was 30 years (range: 2–82 years; IQR: 25–37), and 786 (92%) were male. Because since March 2016, only one in 10 specimens from AHA patients with HIV coinfection have been sampled for HAV sequences, only 166 patients with HAV/HIV coinfection had viral sequencing results, and 161 of those (97%) were infected with the TA-15 strain.

HAV: hepatitis A virus.

The red dot (KX151425) represents the HAV outbreak strain, TA-15. KX15471, KX15472, KX15476 and KX15484 were identified from sewage samples (blue triangles). KX151402, -06, -16, -30, -62 and -69 were from non-outbreak cases (red circles). RIVM-HAV 16–090 and VRD-521–2016 were HAV reference strains from European outbreaks. Cluster groups with bootstrap values greater than 80 are displayed in the figure.

### In-depth investigation, January 2014 to February 2016

From January 2014 to February 2016, we identified 145 outbreak-associated cases and 154 non-outbreak associated cases. In univariate analyses, patients with outbreak-associated AHA were younger (p < 0.0001), more likely to be male (p < 0.0001), and less likely to have been hospitalised (p = 0.01) (Table). About half of the outbreak cases had HIV or syphilis infection, but none of the non-outbreak cases had reported HIV, syphilis or shigellosis infection. Of the 44 (30%) outbreak-associated cases who reported oral-anal sex within 2 months before symptom onset, 29 had casual partners, 18 had more than one partner and 16 had anonymous partners. Infection with the HAV TA-15 outbreak strain was associated with having HIV, syphilis or recent syphilis, gonorrhoea, recent oral-anal sex and with self-identifying as an MSM, whereas non-outbreak HAV infection was associated with recent travel abroad (Table).

**Table:** Characteristics of outbreak and non-outbreak cases, Taiwan, January 2014–February 2016 (n = 299)

Characteristics	Outbreak cases (n = 145)		Non-outbreak cases (n = 154)		Total cases	p value^a^
Median age in years (IQR)	29 (25–34)		33 (28–38)		259	< 0.0001
	n	%	n	%		
Male	144	99	98	64	242	< 0.0001
ALT ≥ 1,000 IU/L	96/144^b^	67	105/152^b^	69	201	0.66
Jaundice	106/141^b^	75	103/153^b^	67	209	0.14
Hospitalisation	79/123^b^	64	108/138^b^	78	187	0.01
Foreign travel within 2 months before symptom onset	20	14	90	58	110	< 0.0001
Use of injection drugs	0	0	1/122^b^	1	1	0.46
Reported HIV infection	75	52	0	0	75	< 0.0001
Syphilis infection any time in the past	80	55	0	0	80	< 0.0001
Syphilis infection in the past year	43	30	0	0	43	< 0.0001
Gonorrhoea infection any time in the past	14	10	1	1	15	0.0002
Gonorrhoea infection in the past year	4	3	0	0	4	0.05
Shigellosis in the past year	3	2	0	0	3	0.11
Oral-anal sex within 2 months^c^ before symptom onset	44	30	0	0	44	< 0.0001
Self-identified MSM^c^	87/144	60	0/98	0	87	< 0.0001

ALT: alanine aminotransferase; HIV: human immunodeficiency virus; IQR: interquartile range; MSM: men who have sex with men.

^a^ p values derived using the nonparametric Wilcoxon rank sum test for medians, and chi-squared test or Fisher’s exact test for proportions.

^b^ Number of patients with information available for this variable.

^c^ Information for male outbreak and non-outbreak cases.

Seventy-four (51%) of 145 outbreak cases were interviewed using a hypothesis-generating questionnaire. Compared with the 45 non-outbreak cases, the proportion eating at sandwich shops or soybean milk shops and consuming salads was higher among the outbreak cases (p < 0.05). However, we did not identify any restaurants or gathering places commonly frequented by the outbreak cases, and the proportion consuming salad ingredients was not different between these two groups.

### Environmental surveillance

**Figure 4:**
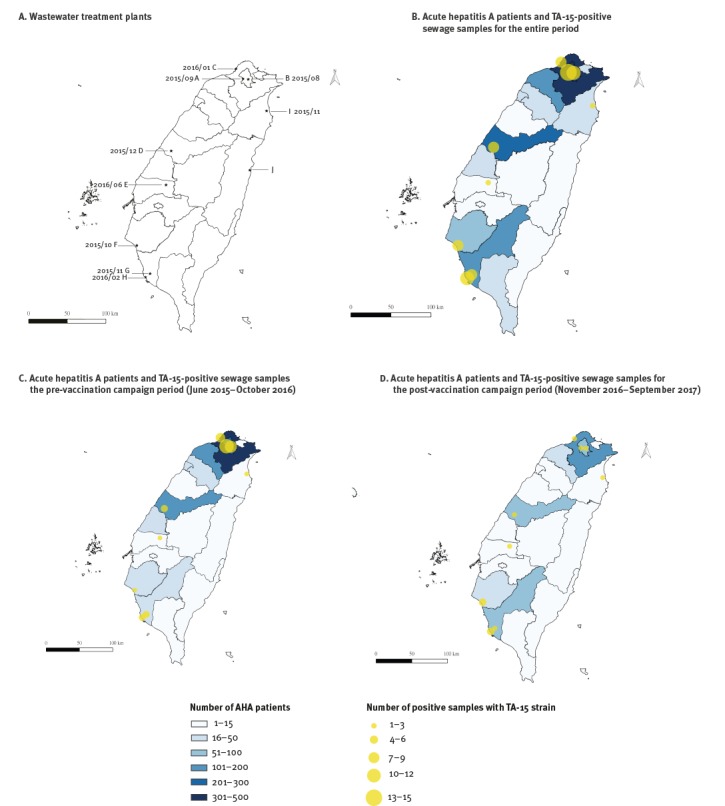
Geographic distribution of wastewater treatment plants, acute hepatitis A patients (n = 1,563) and TA-15-positive sewage samples (n = 74), by county, Taiwan, June 2015–September 2017

AHA: acute hepatitis A; HAV: hepatitis A virus; WTP: wastewater treatment plant;

Sewage specimens were collected twice a month from the inlet collector canals of 10 WTP across Taiwan. Among 540 sewage samples collected during July 2015 to September 2017, 85 (16%) tested positive for HAV. The outbreak strain, TA-15, was first detected in sewage specimens in August 2015 and overall, TA-15 sequences were detected in 74 samples from nine WTP (Figures 2 and 4). We defined June 2015 to October 2016 as the pre-vaccination campaign period, and November 2016 to September 2017 as the post-vaccination campaign period. TA-15 accounted for 17% (53/320) in the pre-vaccination campaign period and decreased to 10% (21/220) in the post-vaccination campaign period. In northern Taiwan, where most AHA patients resided 33 of 53 TA-15 strains were found in the pre-vaccination campaign period, which decreased to six of 21 in the post-vaccination campaign period (Figure 4). The TA-15 strain has not been detected in WTP in August and September 2017.

TA-15 strains were detected in samples collected from WTP A–I, but not WTP J. The dates represent the date of first detection of the HAV outbreak strain TA-15. Free HAV vaccination was provided by the government to close contacts and sex partners of AHA patients in January 2016. The vaccination programme was expanded to other at-risk populations in October 2016.

Blue shading: number of AHA patients in each county; yellow circles: WTP where a TA-15 outbreak strain was detected, with circle size indicating the number of positive sewage samples.

### Phylogenetic analysis

The phylogenetic tree of HAV VP1-2A region was shown in Figure 2. A search using the Basic Local Alignment Search Tool (BLAST) on the National Center for Biotechnology Information (NCBI) website (http://www.ncbi.nlm.nih.gov/) found that the AH2 strain sequence (GenBank accession number: AB020565) was most similar to TA-15, with 99.67% identity (608 nt/610 nt). Based on the maximum-likelihood phylogenetic tree that indicates sequence relationships among Taiwanese HAV, European outbreak strains and other reference strains, the TA-15 grouped together with the RIVM-HAV16-090 (100% nucleotide identity) but not the VRD-521-2016 (95.4% nucleotide identity) outbreak strain in Europe.

### Control measures

The Taiwan CDC provided one dose of free HAV vaccine to close contacts and sexual partners of AHA patients starting in January 2016. In October 2016, the Taiwan CDC expanded the free HAV vaccination programme to individuals born after 1977*, who were living with HIV or newly diagnosed with syphilis or gonorrhoea. Since March 2017, HIV patients who were born before 1977 and seronegative for HAV have also become eligible for free HAV vaccination. By 18 September 2017, 15,487 (62%) of the 24,879 targeted individuals had received at least one dose of HAV vaccine. Information about the HAV outbreak, risk of infection and vaccination was delivered through the media, community-based gay, lesbian, bisexual and transgender health centres and the gay pride parade, as well as physicians providing care for HIV and STI patients. Universal childhood HAV immunisation was implemented through the Expanded Programme on Immunization in 2018.

## Discussion

In June 2015, we identified a continuously circulating HAV outbreak strain in Taiwan that mostly affected MSM and patients with HIV or other STI. In 2016, multi-country HAV outbreaks predominately affecting MSM were also observed in Europe. The EuroPride strain (RIVM-HAV16–090) detected in the Netherlands and other European countries was highly similar to the TA-15 strain [7,15]. A similar outbreak strain was also reported in the United States in 2017 [6], which suggests a global pattern of increased risk among susceptible male adults, with possible transmission through sexual contacts or at MSM events.

Cyclic outbreaks of HAV among MSM or HIV patients have been described in several countries, and outbreak strains among MSM across countries were sometimes closely related and circulated for as long as 10 years [16,17]. Studies have found that people who engage in sex with casual partners, sex in gay saunas, sex with anonymous partners, group sex or oral-anal intercourse, as well as those with household or sexual contact with AHA patients are at increased risk of HAV infection [18-22]. In our investigation, only 30% of outbreak patients reported oral-anal sex before illness onset; the proportion is similar to those with recent syphilis, which might imply recent risky sexual behaviour. Other transmission routes through social networking could be considered but we failed to find any gathering places or social venues that the outbreak cases had in common.

The presence of HAV in sewage could represent evidence of viral circulation in a given community, but HAV genotypes detected in the environment may not consistently correlate with clinical HAV strains detected in concurrent outbreaks in the community [23]. In our survey, the HAV TA-15 strain was detected in sewage samples during the outbreak period, and persistent detection of the outbreak strain was noted in northern Taiwan, where the majority of AHA patients resided. When patients’ residential region gradually shifted from northern Taiwan to southern Taiwan in late 2016 and 2017, we also observed the proportion of positive sewage samples decrease in northern Taiwan. Following the implementation of the HAV vaccine programme in October 2016, the frequencies of both human cases and positive sewage samples decreased substantially.

HAV vaccination is effective for disease prevention and has been used for outbreak control in gay communities [24-27]. Despite the recommendation for HAV vaccination of HIV-infected patients and MSM, vaccination coverage for those at increased risk remains low in Taiwan. A seroprevalence survey in 2009 and 2010 showed that only 10% of MSM aged 18–40 years in Taiwan had anti-HAV antibodies [28]. The cohort effect was noted with increased susceptibly in young HIV-positive patients without nationwide childhood vaccination programme against HAV in Taiwan [29]. A matched case control study showed that having recent STI was associated with AHA among HIV-coinfected MSM [30]. To control the outbreak, we provided free HAV vaccination to at-risk populations, using HIV coinfection or recent STI as an indicator for risky sexual behaviour. The public health authorities contacted HIV patients and patients with newly diagnosed syphilis or gonorrhoea who had been notified by physicians through the NNDSS, and HAV vaccine was provided at hospitals or public health facilities. Although the specific contribution of the vaccine campaign may be hard to quantify as the HAV vaccine programme started after the peak of the HAV outbreak and physicians may have started to promote HAV screening and vaccination when the outbreak was reported, a study in one tertiary hospital HIV cohort demonstrated that the incidence of HAV infection declined after the level of herd immunity exceeded 65% during the ongoing outbreak period in Taiwan [31]. In addition, two recent Australian studies showed that achievement of 40–50% or more than 70% HAV immunity can prevent and control an outbreak [32,33]. Based on the NNDSS and environmental surveillance results, we concluded that the current outbreak was under control once the vaccine programme covered more than 60% of the targeted population. Although HAV vaccination is well tolerated among HIV-infected individuals, the rates of vaccine response and durable protection vary across studies [34-37]. Continuous HAV surveillance and evaluation of long-term vaccine effectiveness among HIV patients is warranted, and universal childhood HAV vaccination will provide more sustainable immunity in the general population.

Our investigation had several limitations. Because of the long incubation period of HAV infection, recall bias may have affected the food exposure history. We therefore interviewed patients using a hypothesis-generating questionnaire to collect exposure information thoroughly. Before this outbreak was recognised, HAV transmission modes other than food-borne may have been ignored, and there may have been under-reporting of sex practices or underdetection of human-to-human transmission. However, the uneven male-to-female ratio and higher prevalence of HIV co-infection in HAV patients was not noted before 2015 in Taiwan [5], and the molecular epidemiology also provides evidence of new circulation of the outbreak strain in the community. Because not all submitted clinical specimens have been tested and sequenced since March 2016, we may have underestimated the true extent of the outbreak caused by the TA-15 strain. Because we started the environmental surveillance in July 2015, unrecognised HAV circulation from subclinical AHA patients in the community in preceding months could be possible.

## Conclusion

We identified an HAV outbreak strain emerging in June 2015 in Taiwan. This HAV outbreak strain mainly affected MSM and patients with HIV or other STI and was controlled with the implementation of an HAV vaccine programme targeting those high-risk populations. We recommend providing HAV vaccination to high-risk populations and conducting continuous surveillance to monitor control measures in community-wide outbreaks of HAV.

## *Authors’ correction

On request of the authors, the names of Pingtung County and Taitung County were corrected in the footnote of Figure 1, and the birth year of people included in the vaccination programme was corrected from 1997 to 1977 in the footnote of Figure 1 and in the chapter on Control measures. These changes were made on 8 April 2019.

## Supplementary Data

149000 Supplement
