# Author’s correction for Euro Surveill. 2019;24(14)

**DOI:** 10.2807/1560-7917.ES.2018.24.15.190411e

**Published:** 2019-04-11

**Authors:** Eurosurveillance editorial team

**Affiliations:** 1 European Centre for Disease Prevention and Control (ECDC), Stockholm, Sweden

On request of the authors, changes were made after publication in the article ‘Outbreak of hepatitis A virus infection in Taiwan, June 2015 to September 2017’ by Chen et al. that was originally published on 4 April 2019: The names of Pingtung County and Taitung County were corrected in the footnote of Figure 1, and the birth year of people included in the vaccination programme was corrected from 1997 to 1977 in the footnote of Figure 1 and in the chapter on Control measures. These changes were published on 8 April 2019.

