# Supplementary material for: Outbreak of hepatitis A virus infection in Taiwan, June 2015 to September 2017
Source: Euro Surveill. 2019 Apr 4;24(14):1800133. doi: 10.2807/1560-7917.ES.2019.24.14.1800133 (PMC6462791; doi:10.2807/1560-7917.ES.2019.24.14.1800133)
Supplement: Supplement [file 18-00133_YANG_Supplement.pdf]

Supplement

Surveillance of AHA in Taiwan

This supplementary material is hosted by Eurosurveillance as supporting information alongside the article [Outbreak of hepatitis A virus infection in Taiwan, June 2015 to September 2017] on behalf of the authors who remain responsible for the accuracy and appropriateness of the content. The same standards for ethics, copyright, attributions and permissions as for the article apply. Eurosurveillance is not responsible for the maintenance of any links or email addresses provided therein.

Environmental Surveillance

Environmental surveillance of sewage sampling was set and in comply with WHO strategy plan of the Global Polio Eradication Initiative in Taiwan in 2012[1]. We collected sewage specimens twice a month from the inlet collector canals of ten wastewater treatment plants (WTPs) across Taiwan. We started to detect HAV in sewage samples in July 2015 for enhanced surveillance.

Amplification and Sequencing of HAV RNA from AHA patients

QIAamp Viral RNA mini Kit (QIAGEN) was used to extract RNA from 140μl of 10% (w/v) homogenate of feces in 1X PBS or 140μl serum and sewage specimens according to the manufacturer's instructions.

cDNA was synthesized and subsequent PCR reaction was generated with SuperScript III One-Step RT-PCR System with Platinum®Taq (Invitrogen). The nested PCR was carried out by SapphireAmp® Fast PCR Master Mix (Takara).The VP1-2A region of HAV genome was amplified with the primers listed in Table 1[2].

One-Step RT-PCR using primers HA021 and HA022 was carried out as described: incubation at 50°C for 30 min, and then at 94°C for 2 min, amplification for 35 cycles, with 1 cycle consisting of denaturing for 30 sec at 94°C, annealing for 30 sec at 53°C, and elongation for 60 sec at 72°C, followed by a final extension at 72°C for 7 min. Nested PCR using HA023 and HA

024 was then performed as described: incubation at 94°C for 2 min, amplification for 35 cycles, with 1 cycle consisting of denaturing for 30 sec at 94°C, annealing for 30 sec at 55°C, and elongation for 10 sec at 72°C, followed by a final extension at 72°C for 7 min.

One-Step RT-PCR using primers HAV-JCT-2F and HAV-JCT-1R-A was carried out as described: incubation at 50°C for 30 min, and then at 94°C for 2 min, amplification for 35 cycles, with 1 cycle consisting of denaturing for 30 sec at 94°C, annealing for 30 sec at 53°C, and elongation for 60 sec at 72°C, followed by a final extension at 72°C for 7 min. Nested PCR using HAV-JCT-2F and HAV-JCT-2R was then performed as described: incubation at 94°C for 2 min, amplification for 35 cycles, with 1 cycle consisting of denaturing for 30 sec at 94°C, annealing for 30 sec at 55°C, and elongation for 10 sec at 72°C, followed by a final extension at 72°C for 7 min.

The nested PCR product was purified with the QIAquick PCR Purification Kit (Qiagen) and used as a template for direct sequencing.

Table 1. Primers used for PCR amplification of HAV DNA.

**Primer name**	**Primer sequence**	**Positions**
HA021[a]	5′-ATT GCA AAT TAY AAY CAY TCT-3′	2904-2924
HA022[a]	5′-TTR TCA TCY TTC ATT TCT GTC C-3′	3430-3451
HA023[a]	5′-CAT TCT GAT GAA TAY TTG TC-3′	2919-2938
HA024[a]	5′-CAT TTC TGT CCA TTT YTC ATC-3′	3420-3440
HAV-JCT-2F[a]	5′-GRA GAA CAG GRA AYA TTC ARA TTA G -3′	2785-2809
HAV-JCT-1R-A[a]	5′-YTT RTC ATC YTT CAT TTC TGT CCA -3′	3429-3452
HAV-JCT-2R[a]	5′-CAG THA RMA CHC CAG CAT CCA T -3′	3378-3399

[a] Beginning and ending points on wild-type HM-175 strain of hepatitis A virus for VP1-2A sequence (Accession: M14707)

Reference

1. WHO. guidelines for environmental surveillance of polio virus circulation. 2003.
2. Ishii K, Kiyohara T, Yoshizaki S, Kawabata K, Kanayama A, Yahata Y, et al. Epidemiological and genetic analysis of a 2014 outbreak of hepatitis A in Japan. Vaccine. 2015;33(45):6029-36.
